# Does Cardiorespiratory Fitness Buffer Stress Reactivity and Stress Recovery in Police Officers? A Real-Life Study

**DOI:** 10.3389/fpsyt.2020.00594

**Published:** 2020-06-23

**Authors:** René Schilling, Christian Herrmann, Sebastian Ludyga, Flora Colledge, Serge Brand, Uwe Pühse, Markus Gerber

**Affiliations:** ^1^Department of Sport, Exercise and Health, University of Basel, Basel, Switzerland; ^2^Zurich University of Teacher Education, Zurich, Switzerland; ^3^Center for Affective, Stress and Sleep Disorders, Psychiatric Clinics, University of Basel, Basel, Switzerland; ^4^Substance Abuse Prevention Research Center, Kermanshah University of Medical Sciences, Kermanshah, Iran; ^5^Sleep Disorders Research Center, Kermanshah University of Medical Sciences, Kermanshah, Iran; ^6^School of Medicine, Tehran University of Medical Sciences, Teheran, Iran

**Keywords:** cardiorespiratory fitness, stress-buffer, cross-stressor adaptation, heart rate variability, occupational stress, stress reactivity, stress recovery, ecological momentary assessment, ambulatory assessment

## Abstract

High levels of cardiorespiratory fitness have the potential to buffer against physical and mental health impairments, which can result from exposure to occupational stress. Police officers are especially at risk of high psychosocial stress; therefore, effective intervention strategies are warranted. Given this background, the purpose of the present study was to examine whether police officers with different levels of cardiorespiratory fitness differ with regard to their (a) physiological stress reactivity during acute real-life stress situations, and (b) physiological recovery related to acute and chronic work stress. In total, 201 police officers took part in this study (M = 38.6 years, SD = 10.1, 35.8% females). Officers were contacted eight times on a smartphone during their workday, and asked to report their current level of positive and negative affect, as well as feelings of stress and anger. Physiological stress responses and recovery (heart rate variability) were assessed using Movisens EcgMove3 devices. The Åstrand bicycle ergometer test was used to assess participants' cardiorespiratory fitness. Chronic work stress was assessed using the effort-reward imbalance model and the job strain model. Multilevel modeling was used to test buffering effects of cardiorespiratory fitness on physiological stress reactivity. Linear regression was applied to test stress-buffering effects of cardiorespiratory fitness on physiological recovery. Results showed lowered physiological stress reactivity to acute work stress in officers with higher levels of cardiorespiratory fitness. However, these results were not consistent, with no effects occurring for feelings of anger, positive affect, and negative affect. Chronic work stress (effort-reward imbalance) was related to lower physiological recovery. Cardiorespiratory fitness was positively related to physiological recovery. Data did not support interactions between work stress and cardiorespiratory fitness on physiological recovery. To some extent, cardiorespiratory fitness seems to have the potential to buffer stress reactivity in police officers in acute stress situations. Therefore, we encourage promoting fitness programs which aim to enhance cardiorespiratory fitness in stressful occupations such as law enforcement. Improvements in cardiorespiratory fitness might further enhance physiological recovery from chronic work stress, which is thought to improve cardiovascular health.

## Introduction

Psychosocial stress is ubiquitous in modern society (1). Although not regarded as negative *per se*, health complaints can occur when individual's coping capacities are exceeded. Documented links between psychosocial stress and health impairments range from physical (e.g. cardiorespiratory) and mental diseases (e.g. burnout) to all-cause mortality (2, 3). Consequently, the individual and societal burden is tremendous (4); hence, health services and researchers are keen to find ways to strengthen coping abilities (5).

Conceptualizations for pathways linking stress to health have historically developed from response (biopsychological) and stimulus (stressor) approaches to transactional processes, in which perceptions of stress play a key role (6). Berntson and Cacioppo (7) argue that mechanisms of stress which affect health involve at least four process components: exposure, reactivity, recovery, and restoration. Exposure is understood as a quantitative representation of perceived stressors. Reactivity refers to the strength of a (physiological) stress reaction in relation to a baseline value. This could be an elevated heart rate following a stress event. Recovery is understood the amount of time required until an individual has returned to baseline level following a stress reaction. Restoration, a more unique concept, refers to “anabolic processes that refresh or repair the organism, because stress may directly impede our ability to perform these functions (e.g., disturbed sleep and impaired wound healing)” (p. 609).

Cardiorespiratory fitness (CF) is understood as a potential buffer in the interplay between stress and health. In their review, Gerber and Pühse (8) gleaned evidence on positive moderation effects for exercise and resulting CF on the interplay between stress and health. Mechanisms of improved cardiovascular health are thought to relate to changes in the autonomic nervous system (8). The sympathetic and parasympathetic nervous system are main components in the sympathoadrenal-medullary (SAM) axis. In stressful situations, the SAM axis pathways increase heart rate, breathing pattern, and blood pressure (9). Recurring and excessive activity of the SAM contributes to increased cardiovascular risk, for example, by increasing the likelihood of hypertension (10). Findings further suggest that physical strain produced by regular engagement in exercise and improved physical fitness are associated with adaptations (i.e. lowered resting heart rate and blood pressure) that may counteract the negative consequences of stress. Furthermore, the cross-stressor adaptation hypothesis (11) suggests that repeated exposure to physical strain can result in at least partially unspecific adaptations, which may cross over to other areas of stress (e.g., psychosocial), and thus lead to more favorable adaptations associated with stress (12).

Research on stress-buffering mechanisms to enhance health has mainly focused on stress reactivity and recovery (2, 8). Previous investigations generally support stress-buffering effects associated with CF (13). However, evidence from three meta-analyses on the impact of CF on cardiovascular reactivity and recovery during and after exposure to experimentally induced stressor tasks have provided heterogeneous results (14–16). In their early work, Crews and Landers (14) showed that CF was associated with a blunted stress reactivity. Twenty years later, Forcier et al. (15) came to a similar conclusion when using more strict inclusion criteria. Thus, their meta-analysis found improved reactivity and some support for improved cardiovascular recovery among individuals with higher CF levels. Controversially, in a further meta-analysis, Jackson and Dishman (16) did not find support for an attenuated stress reactivity among fitter individuals. However, higher fitness levels were associated with a slightly better cardiovascular recovery from laboratory stressors. One strength of their results is the inclusion of studies using submaximal or maximal fitness tests, which, according to the American College of Sports Medicine (17), is a prerequisite for a valid determination of CF. Furthermore, in a recent systematic literature review, Mücke et al. (12) gathered evidence on the influence of CF on stress reactivity in response to the Trier Social Stress Test (TSST), a psychosocial laboratory stressor that has proven to elicit particularly strong stress reactions in previous studies (18). Approximately half of the studies included in this review showed that higher levels of fitness were related to attenuated stress reactivity, as measured *via* heart rate variability and salivary cortisol concentrations (12).

One possible explanation for the inconsistent results in the afore-mentioned meta-analyses might be seen in the diversity of stressors used in the laboratory (19). Artificial stressors are sometimes passive physical performance tasks (holding the hand in a bucket of ice water), and oftentimes consist of cognitive instead of psychosocial challenges. Furthermore, when measuring stress in a laboratory setting, even psychosocial stressors might not be personally relevant (20). Additionally, such stressors are mostly isolated events which are typically short-term, whereas the effects of long-term psychosocial stress are more important from a health perspective. Accordingly, due to limited external validity, results obtained in laboratory settings might not be generalizable to a real-life context. Scholars have therefore emphasized that more meaningful insights should be gained in research carried out in more naturalistic environments (20, 21).

Stress experiences at work are very common in adults (5). Two of the most recognized theoretical models in research on work stress are the effort-reward imbalance (ERI) model, which assumes that stress is the consequence of an imbalance between perceived efforts and rewards at work, as well as the job demands and control (JDC) model, which states that stress occurs if perceived demands and control at work are outbalanced (22, 23). Kivimäki, Virtanen, Elovainio, Kouvonen, Vaananen, and Vahtera (24) showed that in both models increased stress is related to higher cardiovascular mortality. In a retrospective study, the authors examined approximately 800 (mainly male) workers in the metal industry, with an average follow-up time of 25 years. Mortality risk ratios increased to 2.4 (95% CI: 1.3, 4.4) for high ERI, and 2.2 (95% CI: 1.2, 4.2) for high job strain (JDC imbalance), respectively. Furthermore, the risk associated with higher stress levels decreased by 30% in the intermediately physically active group, and by 60% in the highly physically active group.

In order to assess physiological stress reactivity and recovery objectively in real-life, heart rate variability (HRV) has become a popular and frequently used parameter in stress research (25). HRV refers to fluctuations in time intervals of successive heart beats (N-N intervals), measured in milliseconds. These differences can be attributed to branches of the aforementioned SAM axis (sympathetic and parasympathetic nervous system) (26). The Root Mean Squares of Successive N-N Differences (RMSSD), a measure of parasympathetic activity, is used frequently as an indicator of physiological stress reactivity and recovery in real-life measurements (27). Studies assess HRV-based physiological reactivity as hourly (28) and daily (29) aggregations of HRV values, or as the differences between day and night HRV values (29, 30). HRV-based physiological recovery is often assessed as aggregated night HRV levels (28, 29, 31), as well as balance indices between day and night HRV (32). Existing evidence suggests that stress-related differences in HRV are significantly associated with cardiovascular disease (33) and mortality (34).

In their review, Tonello, Rodrigues, Souza, Campbell, Leicht, and Boullosa (35) reported strong negative correlations between work stress and HRV. However, the authors stated that optimal methods for detecting adaptations related to cardiac autonomous stress *via* HRV still need to be developed. A more recent review by Järvelin-Pasanen et al. (27) on work stress and HRV corroborated the general results of Tonello et al. (35), and added more detailed information on the specific HRV parameters that were evidently influenced by chronic and acute work stress. Vrijkotte et al. (32), for example, assessed chronic and acute work stress in a sample of 109 male white-collar workers, which were followed-up for two consecutive workdays. Stress levels were matched to RMSSD, as a HRV parameter reflecting vagal tone. The high ERI group showed lower RMSSD, pointing towards decreased parasympathetic activity in individuals with higher chronic work stress. Work stress of the current day was assessed using the Profile of Mood States (assessed in the evening, retrospectively for the day). Although ERI scores were associated with negative mood states, no significant relationships were observed between mood states and HRV (32). Finally, the randomized controlled trial by von Haaren et al. (28) for the first time points towards the stress-buffering potential of increased CF when objective stress markers are assessed in a real-life context. More specifically, in a sample of 61 sedentary university students, an increase in CF after a standardized physical activity (PA) intervention was associated with higher RMSSD (β = 0.15) during a period of heightened academic stress (exam period).

The existing literature on real-life stress is often based on cross-sectional designs, with single work stress scores or aggregated HRV-values, respectively. Järvelin-Pasanen et al. (27) explicitly highlighted the lack of longitudinal studies. However, studies with longitudinal designs are important for at least two reasons: First, single chronic stress scores or aggregated mean levels in outcomes might show different associations than multiple acute stress perceptions/responses (36). Second, the individual evaluation of the predictability and controllability of stressors is fundamental for the reaction to them (37). Nevertheless, these intra-individual differences are not sufficiently accounted for by inter-individual study designs. In order to highlight differences between situations that are considered highly stressful *versus* not stressful, participants have to function as their own controls. Hence, studies with within-subject designs become necessary (38).

While traditional methods have applied retrospective self-reports, technological improvements enable real-life measurements *via* ecological momentary assessment (EMA). Using portable devices, experiences can be assessed in real-time, rather than necessitating long-term recall (39). Consequently, stress research has experienced a growing interest in state variables, such as emotional reactions and changing mood states (40). Emotions and mood states are understood as cognitively mediated psychophysiological reactions that are limited in time (40). Emotions (i.e. anger, fear) are situational and intense, whereas mood states are longer-lasting, rather unspecific background phenomena, and not consistently cued by specific events (40).

In an ambulatory research study with 73 teachers (*M* = 46.7 years, *SD* = 9.5; 67% male), Pieper, Brosschot, van der Leeden, and Thayer (41) showed independent associations of stressful events and worry episodes with lowered HRV (measured as RMSSD). Uusitalo, Mets, Martinmaki, Mauno, Kinnunen, and Rusko (42) examined the relationship between emotions (stress, irritation, satisfaction) during two workdays using mean values of emotions and HRV in a sample of 19 hospital workers. A strong negative correlation between feelings of stress during work and RMSSD (*r* = −0.70 to *r* = −0.80) indicated lowered parasympathetic activity in an acute stress response. Similarly, chronic work stress, measured as imbalance between efforts and rewards, was strongly related to RMSSD (*r* = −0.53).

Taken together, there is still a marked lack of research addressing the stress-buffering effect of CF in real-life contexts. Therefore, the purpose of the present study is to examine whether police officers with different levels of CF differ with regard to their (a) physiological stress reactivity during acute real-life stress situations, and (b) physiological recovery related to acute and chronic work stress. Based on the aforementioned literature, two hypotheses were tested:

- With the first hypothesis, we expected individuals to show a greater physiological stress reactivity (decrease in HRV) in acute work stress situations with lower group levels of CF (28).
- With the second hypothesis, we expected improved physiological recovery (increased HRV) in groups with higher levels of CF when exposed to acute and chronic work stress situations (30).

## Materials and Procedures

### Participants

Participants were recruited from the police force of the canton Basel-Stadt, a German-speaking city in Switzerland. All employees were invited to participate *via* intranet, internal newspaper, internal TV-adverts, and during team meetings. The present data is part of a voluntary health-check in a bigger project (HERO-study). Additionally, a lifestyle coaching and a second health check were offered to interested officers. Data presented in this article are based on the first health check, which took place between October 2017 and March 2018. Following the invitation, officers had the opportunity to use an e-learning tool, consisting of short text modules and video sequences, in which the general purpose and procedures of the study were explained. Furthermore, information was provided regarding the voluntary nature of participation, the absence of negative consequences in case of non-participation, benefits and risks, rights and obligations, as well as detailed information about the measurements. No monetary incentives were provided to the officers. However, the health check was performed during (paid) working hours and participants received a personalized health profile. At the end of the e-learning phase, participants had the possibility to schedule their own assessment. To participate in the submaximal CF test, participants had to pass a PA readiness check, based on the Physical Activity Readiness Questionnaire (PAR-Q) (43). Participants who did not pass the PAR-Q had to provide a doctor's certificate, attesting that the treating doctor considers it safe for the participant to participate in a submaximal fitness test.

The Basel-Stadt police force consists of approximately 1,000 officers. From these, 227 agreed to obtain more detailed information *via* the e-learning tool (approx. 23%), and 201 officers decided to participate in the study (88%). All participants provided written informed consent prior to data assessment. All procedures followed the ethical principles of the Declaration of Helsinki. The Ethics Committee for Northwest/Central Switzerland approved the study (EKNZ: Project-ID: 2017-01477).

### Procedures

Data used in the present paper was acquired during the laboratory session and in real-life. The laboratory session took place at the education and training center of the police force; all tests were performed in a private setting in the same specially equipped room. All assessments were carried out by the same investigator. In real-life, heart rate was monitored for 48 h. To ensure the assessment of full (daytime) working days among shift-workers, the real-life assessment started on their first day of a shift cycle. By contrast, among participants with regular office shifts, the real-life assessment started on a day before two full working days (usually Monday, Tuesday, or Wednesday). To ensure comparability, we analyzed the full workday, following the first night. Furthermore, this procedure may have served to limit low stress measurements, since participants might have scheduled the laboratory session (health check) on days with relatively low workload. At the end of the laboratory session, participants received the sensors and smartphones, as well as oral and written instructions regarding smartphone usage. The real-life measurement started immediately after the laboratory session. Participants wore the heart rate monitor for 48 h consecutively, and answered questions regarding their stress emotions and affect states on a smartphone (see *Measures* section for more specific information).

### Measures

#### Cardiorespiratory Fitness

Cardiorespiratory fitness (CF) was measured with the validated and internationally applied Åstrand Fitness Test (44). In a submaximal performance test on a bicycle ergometer, maximal oxygen consumption (VO_2_max) is estimated by standardized extrapolations of heart rates at certain resistances (45, 46). Standardized instructions prior to the testing included the avoidance of any strenuous PA for 24 h, as well as heavy meals, and liquids other than water for 3 h. For the test, participants were equipped with a heart rate monitor. Standardized workloads were set for men (150 watts) and women (100 watts). This workload was adjusted to keep the heart rate in the range of 130–160 (bpm) for participants <40 years, and 120–150 (bpm) for participants ≥40 years. Cycling cadence was set at 60 rotations per minute. At the end of each minute, heart rate was noted, and participants stated their perceived exertion on a Borg scale (47). Prior to the test, participants were instructed that perceived exertion should be between 11 to 16 on the Borg scale (below the maximum range) (47). Standardized encouragements were used and participants were controlled for cancelation criteria (17). After 6 min, the test ended if heart rate over the last 2 min did not vary by more than 5 beats per minute. Otherwise, participants were asked to proceed for another minute until this criterion was met. The mean heart rate of the final 2 min was compared against the final stage watts to achieve a gender adjusted VO_2_max (ml/kg/min). These values were matched with CF percentiles using age and gender specific norm values presented by the ACSM (17). Following ACSM recommendations, we further classified participants' CF levels as “very poor,” “poor,” “fair,” “good,” “excellent,” and “superior” (17).

#### Chronic Work Stress

Chronic work stress was measured at the end of the laboratory session. Hereby, the Effort-Reward Imbalance (ERI) scale (22) and the Job Demand and Control (JDC) scale (48) were administered using an online questionnaire. The ERI scale consists of 5 items for efforts (i.e. “I have a lot of responsibility in my job”) and 11 items for rewards (i.e. “I receive the respect I deserve from my superior or a respective relevant person.”). Answers are given on a five-point Likert-scale ranging from 1 (none) to 5 (very high). After summing up each dimension, the ratio was calculated using the factor 0.4545 to counterbalance the unequal number of questions (effort/[reward*0.4545]). Ratios above 1 reflect high levels of job stress (22). The JDC scale consists of five items for demands (i.e. “My job requires me to work very fast”) and six items for control (i.e. “I have freedom to make decisions about my job”). Answers are given on a four-point Likert-scale ranging from 1 (never) to 4 (often). Sum scores for each dimension were calculated. As for ERI, the JDC-ratio was calculated using the factor 0.8333 to balance the unequal numbers of questions (demands/[control*0.8333]). JDC Ratios >1 indicate high work stress (49). The validity and reliability of this instrument has been described previously (50).

#### Ecological Momentary Assessment

Psychological variables in real-life were assessed *via* the EMA method. Each time they were contacted, participants answered different sets of questions on a smartphone (Moto G, 3d Generation) *via* Movisens*XS* (movisens GmbH, Karlsruhe, Germany), an app for Android Smartphones. Movisens*XS* offers a web-based software solution for question settings, sampling contingents, and management of participants; at a later stage, the software processes and prepares data output for analysis. Three sets of questions were triggered in the morning, during the workday, and in the evening. The present paper considers the workday set, which will now be described in detail. The workday set sampled between 12 am and 7 pm for all shift workers (matching their shift schedule) and between 9 am and 5 pm for regular office workers. Sets were time-triggered once per hour with a random appearance of +/− 15 min. The participants responded to an alarm (tone and vibration), which otherwise would repeat every 5 min; if participants did not complete the survey after 15 min, the current assessment was closed. Additionally, participants had the opportunity to postpone the first alarm for up to 15 min. In this case, only one further alarm was triggered 15 min later.

#### Acute Work Stress

In the present study, acute work stress is used as an umbrella term for affective states, as well as feelings of stress, and feelings of anger. The term “feelings of stress” is specifically used for the acute feeling of being stressed, as a single variable only. Approximately 2 min were needed to answer an entire set of questions. Instructions on how to respond to questions associated with the assessment of acute work stress were given during the laboratory session. More specifically, participants were asked to refer to the current situation right before the assessment when answering questions on affect states, feelings of stress, and anger. Since psychological variables were linked to HRV, we controlled for PA as a possible confounder, using two control questions: “Have you been physically active during the past 15 minutes?” and “Have you been physically inactive during the past 15 minutes?” For these questions, participants were instructed to ignore very short walks and standing periods.

Positive and negative affect states were assessed using an adapted German version of the Positive And Negative Affect Schedule (PANAS) (51). Validity of this instrument has been presented previously (52). Participants were asked how they feel at the moment. The items were reflective of five positive (i.e. “content,” “delighted”) and five negative affect states (i.e. “irritable,” “hostile”). Items were answered on a five-point Likert scale, ranging from 1 (not at all) to 5 (very).

Feelings of stress was assessed with a single item: “How stressed do you feel at the moment?” Answers are given on a five-point Likert scale from 1 (not at all) to 5 (very). Validity of single items for the assessment of stress symptoms has been provided previously (53). “Feelings of anger” was assessed with the item “How angry did you get during the last 10 minutes?” Again, items were answered on a five-point Likert scale, ranging from 1 (not at all) to 5 (very).

#### Ambulatory Assessment

Heart rate was assessed using ecgMove3 (movisens GmbH, Karlsruhe, Germany). This sensor records a full one-channel ECG waveform (1024 Hz), 3-dimensional acceleration (63 Hz), and barometric altitude (8 Hz) as raw data on internal memory. Evidence about validity and reliability to accurately capture heart rate and PA has been provided previously (54, 55). At the end of the laboratory session, participants were asked to put on the device, which was worn on a textile dry electrode chest belt.

For heart rate variability (HRV) analysis, two HRV subsets were calculated: (i) 10-min HRV (linked to acute work stress during the workday assessed *via* EMA), and (ii) night HRV (following the workday). We applied the same data processing procedure for both subsets. As mentioned above, the full workday and the following night were considered for data analysis. Accordingly, participants became accustomed to the sensors on the first night. In a first step, the *DataAnalyzer* (movisens GmbH, Karlsruhe, Germany) detected sleep periods and non-wear time (56). In a second step, *UnisensViewer* (movisens GmbH, Karlsruhe, Germany), a software for Windows, was applied to view and edit the data. For calculations of HRV subsets, we stored separate files based on tailored sample limits.

Night HRV sample limits were based on accelerometry sleep detection and set between 8 pm and 8 am. Valid night HRV consisted of at least 4 h of detected sleep, with disruption of no more than 10 min. We assessed all participants individually to detect implausible sleep values. Sample limits for 10-min HRV are taken on the complete workdays, between 12 am to 7 pm for shift workers, and 9 am to 5 pm for workers with regular office shifts. Sample limits begin 10 min before the start times of each acute work stress measurement. We calculated raw interbeat intervals using *DataAnalyzer* (movisens GmbH, Karlsruhe, Germany). The interbeat intervals were exported to Kubios version 3.1.0 (57). Automatic threshold based artefact correction was set at 0.15 s (strong) (57). Frequency-domain parameters are based on Fast Fourier Transformation with frequency bands defined in accordance with the Task Force of the European Society of Cardiology and the North American Society of Pacing and Electrophysiology (58). Welch method was used to calculate spectral parameters (segments 300 seconds, 50% window overlap, 5 Hz Sampling frequency). For further analyses, we extracted time-domain parameter RMSSD.

### Statistical Analyses

Descriptive statistics (M, SD, Range, Skewness, Kurtosis), group differences in outcome variables (t-test, ANOVA), and correlations (Pearson) of outcome variables (HRV night, HRV of the past 10 mi) and predictors (acute work stress, chronic work stress, CF) were calculated using SPSS 26 (IBM Corporation, Armonk NY, USA). For correlations, within subject variables (level 1) were aggregated over the entire day. Distributions of all variables met standards (skewness <2 and kurtosis <7) recommended for parametric testing (59). Univariate analysis of variance was applied to examine group differences in study variables for different CF levels. Probability levels of *p* < 0.05 were interpreted as statistically significant in all statistical analyses.

#### Stress Reactivity (First Hypothesis)

To examine stress-buffering effects of CF on physiological stress reactivity in acute work stress situations, multilevel modelling was calculated with HLM 7.03 (Scientific Software International Inc., Lincolnwood, IL) for Windows. We applied two-level random intercept models. All predictor and outcome variables were standardized (z-scores) (60). HRV (RMSSD), which is nested within persons (level 1), was set as an outcome. CF (6 class equivalent) was set as a predictor on the between-person level (level 2). First, acute work stress variables, which are nested within persons (level 1), were included in both levels, with no significant relations occurring. We then calculated our hypothesized model with preceding acute work stress as the assumed causal variable (level 1), CF (level 2) as moderator, and HRV (level 1) as outcome. Random variations were allowed for predictors; however, interactions were set as fixed effects for causality assumptions. Outcome variables were controlled for gender, age, body mass index (BMI), shift work status, years of service, and PA status during the past 15 min in separate predictor models respectively. With none of these covariates being related to the outcome, they were removed in favor of a parsimonious model. Missing data on level 2 was deleted listwise during calculation. First, the null model and intraclass correlation (ICC = τ_00_/(τ_00_+σ^2^) was calculated for the outcome variable (10-min RMSSD). Second, each predictor variable (level 1: feelings of stress, feelings of anger, positive affect, negative affect; level 2: CF) was included in a separate model. Third, for the between person predictor CF (level 2), four separate models were calculated, each including one of the four acute work stress variables as within-person predictor (level 1). Reported coefficients (pseudo R^2^) refer to estimations with robust standard errors.

#### Stress Recovery (Second Hypothesis)

To examine stress-buffering effects of CF on physiological stress recovery (night HRV), several (four-stage) regression analyses were performed using SPSS 26 (IBM Corporation, Armonk NY, USA). The first stage included all demographic and social background variables, if they were significantly related to the HRV outcome. In the second stage, we entered occupational stress (chronic work stress as ERI ratio and JDC ratio; feelings of stress). Feelings of stress, originally assessed as a within-subject variable, was now aggregated as mean value over the entire workday. The third stage included CF. The fourth stage included an interaction between CF and each work stress variable. In the fourth stage, centered variables of occupational stress and CF were used. We report stepwise changes in R^2^ with the according p-values, and the standardized regression weights with the according *p*-values for each predictor variable in the final model.

## Results

### Sample Description, Group Differences, and Bivariate Correlations

Descriptive statistics are presented in **Table 1**. Overall, 201 officers participated in the study. Participants stating current use of medication (17 participants) or employment status lower than 50% (11 participants) were excluded from data analysis. The remaining sample consists of 115 men (66.5%), and 58 women (33.5%), mean age 37.64 years (*SD* = 9.80), mean years at service 12.32 (*SD* = 8.56). The sample was compared to the entire police corps of Basel-Stadt (*N* = 980 officers), showing significantly younger mean age (*t*-test compared to 41.88 years: *t*(172) = −5.69, *p* < 0.01), but no significant differences in gender ratio (*t*-test compared to 29.6% women).

**Table 1:** Participants' distribution across the six fitness groups and group characteristics in significantly different variables.

Fitness groups based on ACSM standards	*n*	%	Men	Women	Ratio	BMI (*M* ± *SD*)	Heart rate at night (*M* ± *SD*)
Very poor	30	17.3	27	3	90/10	28.58 ± 4.48	74.34 ± 25.12
Poor	13	7.5	7	6	54/46	25.79 ± 4.11	83.39 ± 29.09
Fair	26	15	20	6	77/23	26.00 ± 3.56	83.99 ± 28.61
Good	28	16.2	18	10	64/36	25.94 ± 2.99	64.95 ± 15.91
Excellent	25	14.5	13	12	52/48	24.66 ± 2.33	73.11 ± 30.11
Superior	51	29.5	30	21	59/41	24.64 ± 2.64	66.56 ± 23.15

ACSM, American College of Sports Medicine; BMI, Body Mass Index.

Five participants (2.7%) did not answer any acute work stress assessments. Additionally, 13 (7.5%) acute work stress datasets were missing due to software problems (*n* = 7, 4.0%) or invalid data on heart rate variability (*n* = 6, 3.5%). On average, participants answered 6.47 (*SD* = 1.34) acute work stress measurements (of a possible 8) over the course of the study. Following Lüdtke, Trautwein, Kunter, and Baumert (61), we calculated ICC(1)^1^ for within-level and ICC(2)^2^ for between-level reliability of the psychometric measures. ICC(1) can be interpreted as the percentage of variance that can be accounted for by differences between persons. ICC(1) values were 0.19 (19%) for anger, 0.45 (45%) for stress, 0.67 (67%) for positive affect, and 0.61 (61%) for negative affect. Applying ICC(1) to the Spearman Brown formula, we calculated ICC(2) to estimate the accuracy of the mean values across all individuals. ICC(2) values were 0.60 (moderate) for anger, 0.84 (good) for stress, 0.93 (excellent) for positive affect, and 0.91 (excellent) for negative affect (62). Missing data in night heart rate parameters occurred for 17 participants (9.8%), whereas five (2.9%) datasets were excluded due to artefacts and in 12 cases (6.9%), sensors did not provide data for unknown reasons.

The overall mean of fitness percentiles for CF was 62.93 (*SD* = 33.37). The participants' distribution across the six-group classification recommended by the ACSM is described in **Table 1**. Group values and standard deviations are presented for variables in which the groups differed significantly. Significant differences occurred for gender ratio (*F*(5,167) = 2.81, *p* < 0.05), body mass index (*F*(5,167) = 6.13, *p* < 0.01), and heart rate at night (*F*(5,150) = 2.45, *p* < 0.05). No significant between-group differences were found for age, shift work status, years of service, HRV parameters at night, and any acute or chronic work stress variables. Interestingly, the very poor fitness group showed lower levels of heart rate at night compared to the poor and fair fitness group. However, *post-hoc* tests did not reach significance (Tukey, Bonferroni).

The bivariate correlations between the different study variables are presented in **Table 2**. These findings show that CF levels (percentiles) were positively related to the aggregated mean values of HRV during the workday. The two chronic work stress questionnaires (JDC, ERI) were positively correlated with each other. While the JDC ratio was only correlated with the acute work stress measure feelings of anger, the ERI ratio was similarly associated with feelings of stress, feelings of anger, and negative affect. Acute work stress parameters and the JDC ratio were not significantly related to HRV outcomes. By contrast, the ERI ratio was negatively associated with night HRV.

**Table 2:** Descriptive statistics for and bivariate correlations between independent and dependent variables.

Variable	Range							Bivariate correlations between the study variables								
	*n*	*M*	*SD*	Min	Max	Skew	Kurt	1.	2.	3.	4.	5.	6.	7.	8.	9.
1. Cardiorespiratory fitness class	173	45.34	11.40	21.90	89.40	0.66	0.71	–								
Chronic work stress																
2. Job Demand Control ratio	162	0.96	0.19	0.54	1.55	0.77	0.46	0.13	–							
3. Effort Reward Imbalance ratio	162	0.89	0.25	0.33	2.02	1.03	2.33	-0.03	0.28**	–						
Acute work stress (aggregated mean values per workday)																
4. Feelings of stress	155	1.70	0.62	1.00	3.86	0.96	0.48	-0.04	0.08	0.28**	–					
5. Feelings of anger	155	1.61	0.52	1.00	3.20	0.77	0.04	-0.03	0.21**	0.20*	0.66**	–				
6. Positive affect	155	16.50	2.57	5.86	23.00	-0.37	1.33	0.00	-0.12	-0.13	-0.34*	-0.15	–			
7. Negative affect	155	7.58	2.38	5.00	15.71	1.13	0.95	0.00	0.13	0.28**	0.74*	0.58**	-0.39**	–		
Heart rate variability																
8. Night RMSSD	156	45.81	17.32	10.60	98.84	0.85	0.60	0.08	0.13	-0.29**	-0.85	0.08	0.04	-0.05	–	
9. 10-min RMSSD	154	34.70	11.05	5.00	75.00	0.24	0.64	0.17*	0.15	-0.02	-0.09	0.01	0.03	-0.03	0.40**	–

RMSSD, Root Mean Squares of Successive N-N Differences.

*p < 0.01; **p < 0.001.

### Multilevel Model to Examine Physiological Stress Reactivity (First Hypothesis)

Results of the multilevel modelling are presented in **Table 3**. The intraclass correlation provided evidence for a two-level hierarchical structure, showing that 63% of variance in the outcome can be accounted for by intra-individual variables (RMSSD ICC = 0.63) (63). Results of multilevel models are described for CF and acute work stress (gender, age, BMI, shift work status, years of service, and PA during the past 15 min controlled for in separate models; see *Methods*). In accordance with the hypothesis, a cross-level interaction for CF suggests a moderation effect, with higher levels of RMSSD being predicted by higher levels of CF when participants perceived stronger feelings of stress. On the contrary, no interaction effect occurred for feelings of anger, positive affect, and negative affect. Between-subject differences (level 2) in CF were a significant predictor of 10-min RMSSD during the workday. The direction of the association of feelings of stress, as a within-subject (level 1) predictor, and 10-min RMSSD was negative, but not statistically significant. Surprisingly, 10-min RMSSD during the day was not significantly affected by the acute work stress measures.

**Table 3:** Estimated effects in multilevel models using restricted maximum likelihood with predictors CF, feelings of anger, positive affect, and negative affect on HRV outcome variable RMSSD over the past 10 min.

Outcome	Null model				Level 2				Level 1				Full model							
													Level 1				Cross-level interaction			
	β	*SE*	*T*	*p*	β	*SE*	*T*	*p*	β	*SE*	*T*	*p*	β	*SE*	*T*	*p*	β	*SE*	*T*	*p*
					Cardiorespiratory fitness class				Feelings of stress								Cardiorespiratory fitness class × feelings of stress			
10-min RMSSD	0.03	0.07	0.52	0.751	0.14	0.07	2.00	0.045	-0.02	0.03	-0.51	0.437	-0.03	0.03	-1.02	0.324	0.06	0.03	2.04	0.043
									Feelings of anger								Cardiorespiratory fitness class × feelings of anger			
									-0.02	0.02	-0.69	0.407	-0.02	0.02	-0.69	0.433	-0.00	0.03	-0.69	0.372
									Positive affect								Cardiorespiratory fitness class × positive affect			
									-0.06	0.04	-1.34	0.357	-0.06	0.04	-1.34	0.397	-0.01	0.05	-0.15	0.469
									Negative affect								Cardiorespiratory fitness class × negative affect			
									0.03	0.03	0.83	0.395	0.03	0.03	0.83	0.375	-0.00	0.03	-0.00	0.148

RMSSD, Root Mean Squares of Successive N-N Differences.

### Regression Analyses to Examine Physiological Stress Recovery (Second Hypothesis)

The results of the regression analyses are provided in **Table 4**. In all models, age significantly explained night HRV levels, with lower levels of HRV in older individuals. ERI significantly explained variance in night RMSSD. The JDC ratio and feelings of stress did not significantly predict night HRV. CF significantly explained variance in participants' night RMSSD levels, after controlling for feelings of stress. Finally, the interaction terms did not significantly account for variance in the outcomes in any of the calculated models.

**Table 4:** Linear regression analyses predicting night RMSSD with occupational stress and cardiorespiratory fitness.

	Δ*R*^2^	β	*p*	Δ*R*^2^	β	*p*	Δ*R*^2^	β	*p*
**Step 1**	0.29		0.000	0.29		0.000	0.31		0.000
Years of service		-0.01	0.905		-0.01	0.905		-0.01	0.947
Age		-0.51	0.000		-0.51	0.000		-0.52	0.000
Shift work		-0.05	0.553		-0.05	0.553		-0.07	0.381
**Step 2**	0.00		0.384	0.02		0.048	0.00		0.872
Occupational stress									
JDC ratio		0.07	0.384						
ERI ratio					-0.14	0.048			
Feelings of stress								0.00	0.872
**Step 3**	0.02			0.02		0.006	0.03		0.002
Cardiorespiratory fitness class		0.12	0.006		0.12	0.006		0.17	0.002
**Step 4**	0.00		0.839	0.00		0.902	0.01		0.092
Occupational stress ×		-0.04	0.839		0.01	0.902		0.11	0.092
cardiorespiratory fitness class									

JDC, Job Demand Control; ERI, Effort Reward Imbalance; RMSSD, Root Mean Squares of Successive N-N Differences.

## Discussion

The aim of the present study was to examine the stress-buffering effects of cardiorespiratory fitness (CF) under realistic conditions. CF significantly moderated physiological stress reactivity (in RMSSD) when participants perceived elevated feelings of stress at work, with higher levels of CF predicting higher levels of parasympathetic activity. However, no direct effects occurred for perceived feelings of stress, feelings of anger, positive affect, or negative affect. Interestingly, the physiological stress reactivity was not significantly affected by acute psychological work stress. Chronic work stress, measured as effort reward imbalance (ERI), was negatively related to physiological recovery at night. While CF was associated with increased physiological recovery at night, no effects on recovery appeared for interactions between CF with chronic and acute work stress. The present pattern of results adds to the current literature in an important way, as one of the first real-life studies examining stress-buffering effects of CF in police officers. With these results, we add distinct insights into the influence emotional and affective states. Our results are novel, because the study design and statistical analysis further accounts for intra-individual differences in the stress response.

With our first hypothesis, we expected stress-buffering effects of CF on HRV in acute work stress situations. CF showed the expected effect when feelings of stress increased, with lower physiological stress reactivity (higher parasympathetic activity) in more fit individuals. The present results corroborate previously proposed stress-buffering effects of CF on cardiovascular stress reactivity (12, 15, 28). However, these results only appeared for feelings of stress, whereas no effects appeared for feelings of anger, and affective states. Since affective states are understood to be less cognitively presented in stress perception, emotions might be more closely related to acute work stress (40). The missing effects for feelings of anger might be related to individual differences in adaptive pathways in the regulation of the stress response (64). As mentioned in the introduction, cognitive processes are key ingredients in the emergence of stress. Anger is linked to a cognitive appraisal (65) of causality and responsibility (66), for example when another person is (perceived to be) responsible for an event with negative consequences for oneself (66, 67). This accountability can discriminate anger from other emotions, such as fear or anxiety, when attributions are not possible (67, 68). Thus, stress can even predispose an individual towards anger (65). However, accountability is, by definition, related to higher perceived controllability and predictability of events, which are key factors in the stress process (37). In line with this, Wu, Gu, Yang, and Luo (69) showed that anger was associated with a higher HRV (higher parasympathetic activity) compared with fear. Consequently, a relatively low reactivity to anger, compared to other emotions, would result in weaker associations, in line with the anticipated buffering effects of CF. In sum, theoretical assumptions and evidence regarding ANS reactions encourage a differentiated, nuanced contemplation of the emotions referred to here (70, 71). Furthermore, no direct effects of acute work stress appeared on HRV. This result is somewhat counterintuitive. In their systematic review, Jarczok et al. (72) showed that adverse psychosocial work conditions were associated with lowered HRV. However, in a 3-day EMA study by Kamarck, Muldoon, Shiffman, Sutton-Tyrrell, Gwaltney, and Janicki (73), the association between demands and physiological parameters reflecting cardiovascular health was not limited to the workplace. Further research is needed to further examine the influence of psychosocial stress during leisure time.

While the present sample represented the overall gender ratio in the police corps, it was also significantly younger. Due to some promising results in older populations (32, 74), our findings might understate possible relevant health effects. Furthermore, police officers have to take physical examinations in the early stages of their career; hence, relatively high CF levels can be expected among study participants. As a consequence, the detected effects of CF as a stress-buffer might be rather conservative in the present study.

With our second hypothesis, we expected improved stress-related physiological recovery patterns (increased parasympathetic activity in high stress levels) with higher levels of CF. Although chronic work stress (ERI) showed negative associations with night HRV, no support of stress-buffering effects appeared for CF. However, CF predicted improved physiological recovery for night RMSSD. Interestingly, chronic work stress measured *via* the JDC ratio, and acute work stress, were not related to night HRV. These results are partly in line with the findings of two reviews (27, 75). Loerbroks et al. (75) found significant age-related partial correlations with ERI and HRV, but not JDC in the age group of 35 to 44 years. The review of Järvelin-Pasanen et al. (27) contained five studies assessing RMSSD. Of these studies, two studies unanimously reported lower RMSSD levels in stressed individuals. However, two studies only found partial support, whereas in one study no significant effect of chronic work stress on RMSSD was detected (76). Interestingly, in the real-life study by von Haaren et al. (28), the control group showed lower levels of night RMSSD during the examination period, compared to the intervention group. These previous results support the present finding, that chronic work stress is associated with reduced physiological recovery processes, measured as HRV.

One explanation for these results might be related to the components of the stress-health relationship (7). As mentioned previously, research on stress-buffering effects of CF mainly focus on physiological stress reactivity and recovery. Nevertheless, with the present paper, we want to encourage researchers to place a stronger focus on restoration processes in future investigations. The descriptions by Berntson and Cacioppo (7) indicate that CF might be related to improved cardiac vagal activity (parasympathetic influence on cardiovascular stress reactivity and recovery) due to anabolic processes. These adaptations might be rather long-term, hence, health-related improvements might be less affected by short-term changes in stress levels. The present study supports this notion, since chronic work stress levels showed more consistent associations with favorable HRV patterns than acute work stress. Interestingly, CF levels did not consequently predict night HRV in all models, whereas mean levels of heart rate at night differed significantly between CF groups. Hence, CF might have different influences on heart rate and HRV (8, 77). These differences are important to understand, since high heart rate and low HRV have been shown to be independent risk factors for cardiovascular disease (33, 41, 78).

### Strengths

One of the key strengths of this study is constituted by the methodological advancements in relation to previous research. With the application of real-time assessments of stress reactivity, and with the co-assessment of physiological and psychological parameters, we tackled several gaps in previous research. For instance, using EMA techniques has the potential to minimize bias related to recall times, which constitutes a huge improvement in allowing the examination of the “experiencing self” in contrast to the “reflecting self” (79). Furthermore, our statistical analyses took into account the individuality of the stress-health relationship by statistically accounting for intra-individual changes in stress perceptions and reactions. A further strength of our study is the improved quality of night HRV assessment by including an additional night of acclimatization. Additionally, the present results are based on a rigorous assessment of study variables, with standardized, reliable, and valid tools that are widely applied in international research. The homogeneity of the present sample further allows cohort-specific interpretations in a highly stressful occupation.

### Limitations

However, some limitations may affect the generalizability of our data. First, we tested stress reactivity and recovery independently. However, some scholars suggest that these phenomena are interdependent. The “*DynAffect*” model by Kuppens, Oravecz, and Tuerlinckx (80) states that stress responses are dynamic, fluctuating around an individual's emotional “home base.” In this sense, reactivity and recovery are understood as the sensitivity to withdraw from, and the attractor strength that ties back to, the home base (80). Furthermore, Smyth, Sliwinski, Zawadzki, Scott, Conroy, Lanza (81) recently introduced their stress response assay that comprises both stress reactivity and recovery. The assay additionally captures pile-up, which accounts for multiple stress responses within a defined time-period. Second, controlling for extraneous variables was not feasible, given the nature of our study design. However, using real-life stressors increases the ecological validity of our findings. Third, although HRV assessments followed the recommendations of the Task Force of the European Society of Cardiology and the North American Society of Pacing and Electrophysiology (58), breathing patterns and physical activity were not controlled for objectively. To improve the quality of subjective physical activity reports, all participants received verbal and written instructions prior to the assessments. Fourth, our results might include errors due to multiple testing on the same dataset. With regards to the associated effect sizes and *p*-values, our results must be interpreted with caution. In this respect, future observations in police officers may benefit from larger sample sizes.

### Practical Implications

Occupational stress among police officers may have a more direct impact on society than that of other occupations, since job performance is closely linked to public safety (82). However, police officers have been shown to be at risk for maladaptive coping strategies (83). Associated organizational costs are considerable due to reduced productivity, absence, and early retirement (84). In this respect, self-regulatory processes to manage emotions and sustain resilience are highly important (85). Self-regulatory techniques primarily aim for an efficient systemic recalibration to physiological and psychological balance after intense stress experiences (85). Furthermore, successful recovery from stress is associated with more favorable physiological stress markers, i.e. cortisol, which is evidently linked to improved functioning of higher-order cognitive tasks. Optimal brain functioning has been related to enhancements in concentration, planning abilities, memory, decision making, moral reasoning, inhibition of inappropriate responses, and inhibition of distractions (85).

Applying HRV biofeedback to successfully enhance coping is well-documented (86). Firstly, monitoring the physiological stress reaction could increase self-awareness of stress experiences (86). Secondly, specific techniques which enhance the regulation of physiological function can be learned (86). These improvements in the physiological stress reaction can complement further psychological self-regulatory techniques (85). In the present results, partial support appeared for CF as a physiological resilience factor, helpful for managing stress in police officers.

Hence, one potential way for police health authorities to enhance self-awareness is to encourage employees to monitor day and night values of HRV. We further emphasize the importance of interventions that focus on improved CF. The present results have shown the association of increased fitness and several known risk markers of cardiovascular health.

## Conclusion

Our results showed partial support for the potential of CF to buffer cardiovascular reactivity when police officers are exposed to acute work-related stress. Higher levels of CF were related to enhanced physiological recovery, which might have further important implications for participants' health. Therefore, we encourage the promotion of fitness programs with the aim of enhancing CF in stressful occupations such as law enforcement. Finally, we encourage the assessment of HRV for the early detection of maladaptive acute physiological stress reactivity, as well as physiological recovery related to chronic work stress.

## Data Availability Statement

The datasets generated for this study are available on request to the corresponding author.

## Ethics Statement

The studies involving human participants were reviewed and approved by Ethics Committee for Northwest/Central Switzerland (EKNZ: Project-ID: 2017-01477). The patients/participants provided their written informed consent to participate in this study.

## Author Contributions

RS, SB, and MG made substantial contributions to conception and design of the study. RS, SL, and MG were responsible for the acquisition of data. RS, CH, and FC were responsible for the analysis and interpretation of data. RS and CH drafted the manuscript. FC, SB, MG, and UP wrote sections of the manuscript. CH, SB, SL, UP, MG, and FC critically reviewed and revised the initial draft. All authors contributed to the article and approved the submitted version.

## Conflict of Interest

The authors declare that the research was conducted in the absence of any commercial or financial relationships that could be construed as a potential conflict of interest.

## Footnotes

1 ICC(1) = τ2/(τ2 + σ2)

2 ICC(2) = k × ICC(1) / (1 + (k − 1) × ICC(1)
